# Supplementary figures and images for: Extracellular spreading of Wingless is required for Drosophila oogenesis
Source: PLoS Genet. 2021 Apr 2;17(4):e1009469. doi: 10.1371/journal.pgen.1009469 (PMC8046344; doi:10.1371/journal.pgen.1009469)

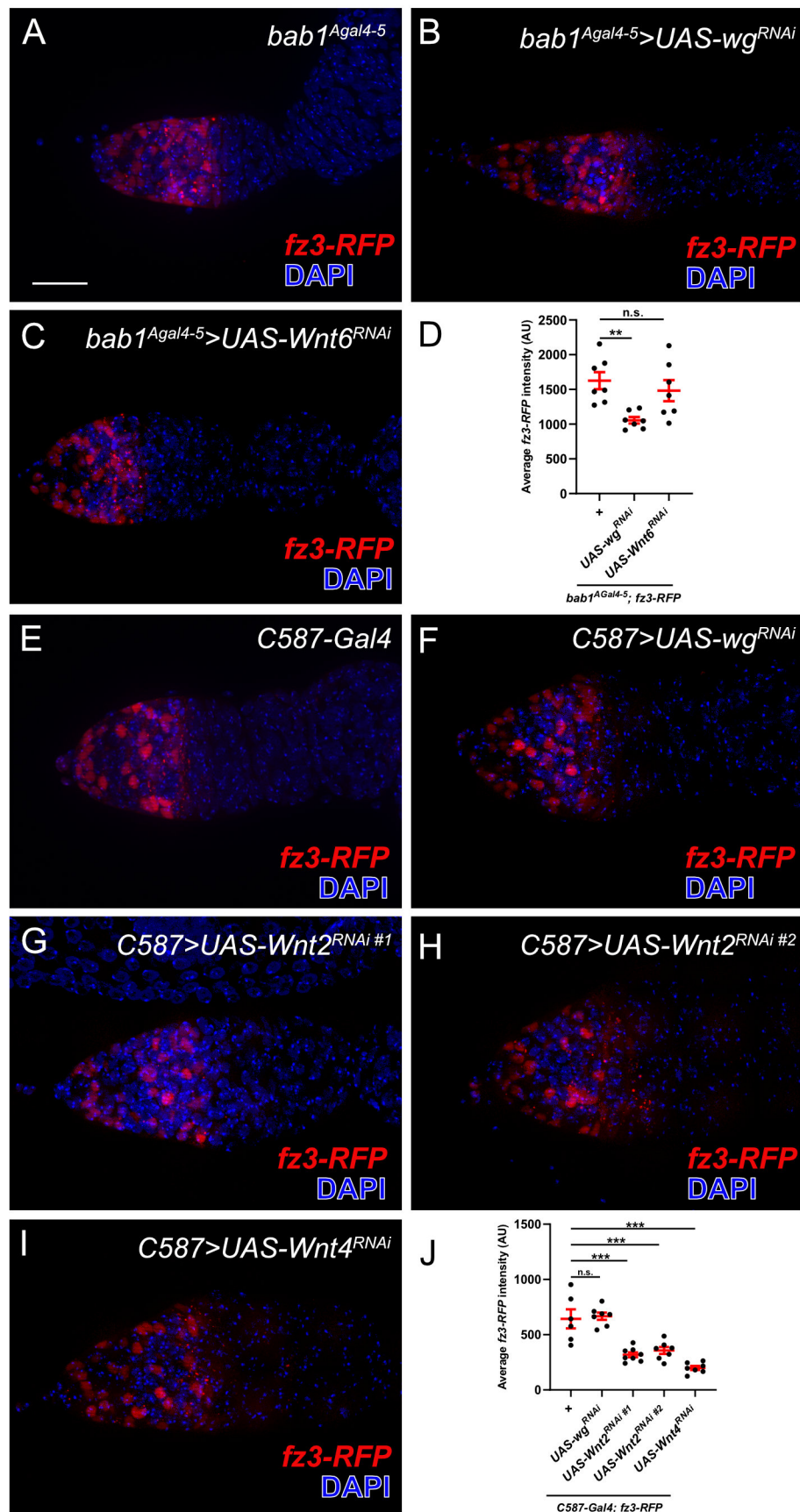

Supplement: S1 Fig — A-J. The Wnt signaling reporter fz3-RFP in red, nuclei stained with DAPI in blue. Scale bar: 20 μm. A-C. bab1Agal4-5, expressed in cap cells and terminal filament cells, was used to knock down wg (B) or Wnt6 (C), both expressed in cap cells. D. Quantification of fz3-RFP intensity. Knockdown of wg resulted in decreased fz3-RFP expression, whereas knockdown of Wnt6 did not affect fz3-RFP expression. E-I. C587-Gal4, strongly expressed in escort cells, was used to knockdown wg (F), Wnt2 (G,H) or Wnt4 (I). Wnt2 and Wnt4 are expressed in escort cells. J. Quantification of fz-RFP intensity. Levels are comparable in controls and when wg is knocked down in escort cells, consistent with previous findings that wg is expressed in cap cells, not escort cells. Knockdown of Wnt2 in escort cells using two independent RNAi lines or knockdown of Wnt4 in escort cells results in decreased fz3-RFP expression. ** indicates p value: 0.0099–0.001, *** indicates p value ‌<0.00099, n.s. indicates not statistically significant. Each dot in D and J indicates average RFP intensity for a single germarium. n = 6–8 germaria per genotype. AU: Arbitrary Unit. (PDF) [file pgen.1009469.s001.pdf]

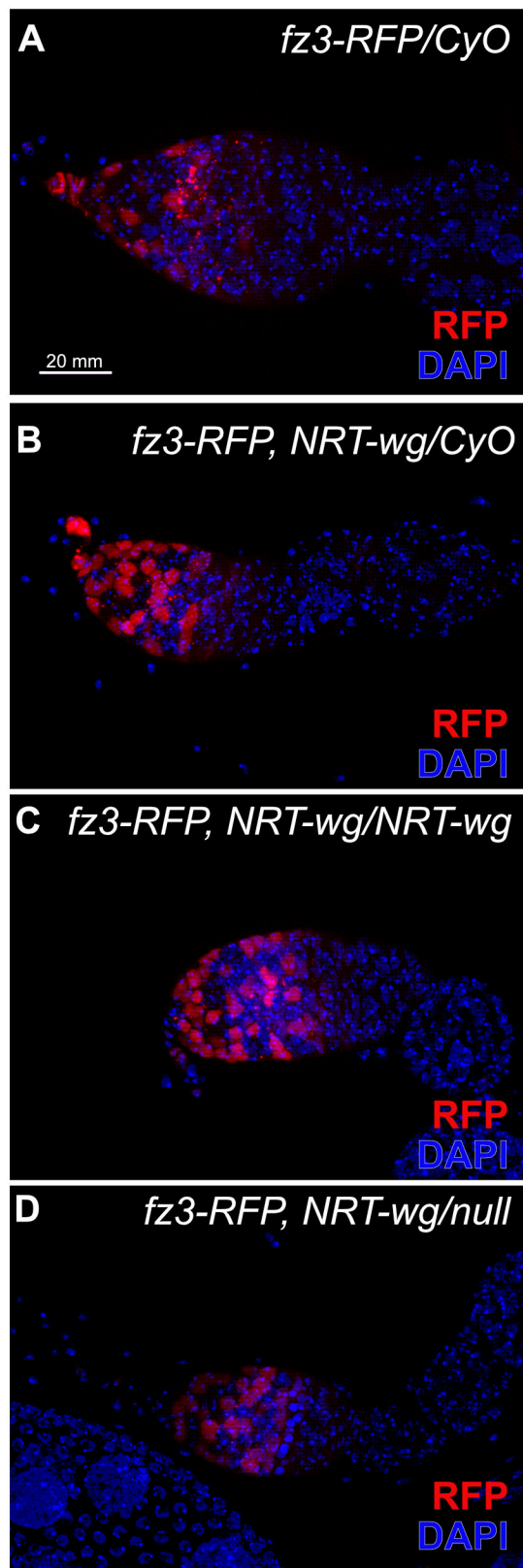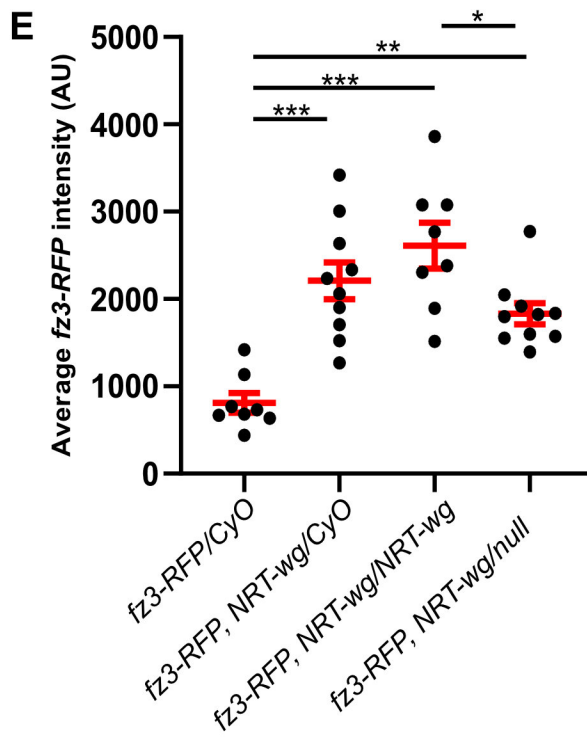

Supplement: S2 Fig — A-D. A comparison of fz3-RFP expression in the germaria of (A) control (B) NRT-wg heterozygous, (C) NRT-wg homozygous, and (D) NRT-wg/null flies shows that membrane tethering of Wg unexpectedly induces fz3-RFP expression in germaria. DAPI labels nuclei. AU: Arbitrary units. Scale bar: 20 μm. E. Quantification of fz3-RFP intensity in germaria of the indicated genotypes. Each dot indicates average fz3-RFP intensity in one germarium. n = 8–10 germaria per genotype. * indicates p value: 0.01–0.05, ** indicates p value: 0.0099–0.001, *** indicates p value ‌< ‌0.00099. (PDF) [file pgen.1009469.s002.pdf]
