# Supplementary material for: Extracellular spreading of Wingless is required for Drosophila oogenesis
Source: PLoS Genet. 2021 Apr 2;17(4):e1009469. doi: 10.1371/journal.pgen.1009469 (PMC8046344; doi:10.1371/journal.pgen.1009469)
Supplement: Attachment — Submitted filename: Response to reviewers 12-4.docx [file pgen.1009469.s004.docx]

**Reviewer #1:**

*This manuscript is framed as answering the question of whether extracellular spread of Wg ligand is functionally important in the ovary. One motivation is the report a few years ago that membrane-tethered Wg can support wing development despite prior evidence of morphogen action from a localized source; another stems from a more local dispute about sources and spread of Wnt ligands in the germarium. The authors present some evidence that membrane-tethered Wg does not substitute for normal Wg in the germarium. However, for both the more general purpose of presenting a strong paradigm for functional extracellular spread of Wg and further definition of Wnt signaling roles in the germarium, rigorous and detailed evidence is essential. There appear to be important gaps in the evidence presented.*

The reviewer correctly articulates two of the motivations of our study. Yet a third motivation for us is testing our model of Dlp function, as described in our 2014 paper: that Dlp is required for Wnt spreading to the follicle stem cells. This model makes a clear prediction about follicle stem cell proliferation in the tethered wingless flies, which can be tested with NRT-wg.

*Genetic considerations:
The authors acknowledge that it is important to limit Wg manipulations to the tissue and time period studied, and that they were unable to implement an ideal test. The next best test was to restrict Wg/Nrt-Wg exchange to adulthood. However, very few results are shown for such flies. Of prime importance is the most definitive measure of the presumed major deficit- FSC division and to pick key times, where Wg signaling was clearly altered but little time passed subsequently to allow for secondary consequences to develop (the 10d period at 29C used seems likely to allow secondary phenotypes). It is also not clear whether these flies were homozygous or hemizygous for the altered allele (both would likely be informative).*

We have now clarified in the manuscript that the adult-converted flies (wg 🡪 NRT-wg) are hemizygous for the *wg* or *NRT-wg* allele. As shown in new Fig. 1A, the homologous chromosome carries the *Gal4* construct inserted in the *wg* KO locus, so it is null for *wg*. Without significantly changing the conversion strategy, it is not possible to convert both chromosomes. Fortunately, converting one copy in a hemizygous background seems to be the better experiment because homozygous *NRT-wg* would likely introduce increased toxicity from the tethered-Wingless protein (as we discuss below).

In response to the reviewer’s request, we have now measured follicle stem cell proliferation in the adult-converted flies and reported that data in Fig. 3, and for this experiment we assayed the proliferation rate 5 days after the temperature-shift to 29°. We still wait 5 days because the experiment requires 1) all (or nearly all) the *wg*-expressing cells to undergo FLP-mediated recombination after Gal4 induction, 2) the existing Wg protein to be turned over, and 3) the cells to alter their behavior in response to the loss of Wg protein.

*The phenotype of flies with some form of Nrt-Wg allele includes a component which appears unfortunately to be of unknown origin or defined consequence. That is exemplified here mainly by comparison of homozygotes and hemizygotes but was also apparent in the original Nrt-Wg allele publication, including some notes on significant differences with and without markers downstream of the Nrt-Wg coding region. Hence, there is some uncertainty concerning the contribution of completely unknown factors, effects possibly related to Nrt-Wg expression levels and effects which might be dominant (hypermorphic or neomorphic). These concerns are brought up by the authors with regard to homozygotes. However, it seems to me that Nrt-wg/+ flies should be used for comparisons in all assays. Nrt-wg/ wg-ts would also seem to be an attractive combination for an alternative way of looking at adult-onset phenotypes because the control with altered temperature conditions would be genetically identical.*

We performed further experiments to characterize the the *NRT-wg* allele and have included them in Fig. 1. (We describe them in response to reviewer 2, who suggested them.) As Reviewer 1 notes, the methods section of the 2014 original tethered-wingless paper described unpublished data that the excision of downstream marker cassettes in the *NRT-wg* flies improved their viability; they concluded, “Our evidence so far is consistent with the notion that the developmental delay of *wg{KO; NRT–Wg}* animals could be due mostly to a mild reduction in expression and hence activity” (Alexandre et al, 2014 supplementary methods). In contrast to Alexandre’s unpublished data, the data in our paper show that developmental delay of *NRT-wg* animals is reduced when the dose of *NRT-wg* is reduced, implying that *NRT-wg* is toxic and can be tolerated better when the level is reduced.

With respect to the best controls, that will depend on the experiment. We have provided a number of controls for the survival data, including *NRT-wg/+* as requested by the reviewer (new panel 1C); for visualizing Wg protein, here the best comparison is *NRT-wg/NRT-wg* compared to +/+ because the dose of Wg protein is the same; for the adult conversion experiments, it is not possible to provide the *NRT-wg/+* control because of the need for *wg-Gal4.*

*Measurements:
There are two major deficits.
First, I cannot understand why the authors do not present images of Fz3-RFP in the various conditions tested. These authors previously verified Fz3-RFP as an excellent marker of Wnt signaling in the ovary and they are well aware of the presence and contributions of other Wnts (which they have tested). The expectation, according to their model, is that Fz3-RFP will be drastically reduced when Nrt-Wg replaces Wg. That is important to show but it will also be interesting to see what remains (and how quickly Fz3-RFP declines in conditional allele tests). For readers without prior knowledge of the germarium it is important to point out that other Wnt ligands are present and presentation of Fz3-RFP will facilitate that discussion.*
As the reviewer states, we previously determined that Fz3-RFP is a reporter of Wnt pathway activation, but it was unknown which Wnt ligand was responsible this activation. We have now rigorously assessed the expression of the Wnt pathway reporter Fz3-RFP in germaria when each of the four Wnt ligands expressed in the germarium is knocked down. We find that Fz3-RFP levels are modestly reduced when *Wnt2, Wnt4,* or *Wg* is knocked down, suggesting that it responds to many endogenous Wnt ligands (see new supplemental figure S1). Unfortunately its lack of specificity suggested it would not be useful as reporter to test predictions about NRT-wg.

Nevertheless, to meet the reviewer’s request and determine the effect of *NRT-wg* on Fz3-RFP, we had to generate a recombinant between *NRT-wg* and the unmapped Fz3-RFP reporter. Unfortunately, we discovered they are very tightly linked (and subsequent experiments seem to suggest that NRT-wg may inhibit recombination, see below), so the recombination required a molecular assay and took many months. After finally generating the fly, we were surprised to find that Fz3-RFP levels are not reduced but increased with NRT-wg, suggesting some kind of relay between Wg ligand near the cap cells and Wnt signaling in the escort cell region, perhaps via Wnt2 and/or Wnt4 (see new supplemental figure S2). Thus, Fz-RFP is a complex reporter for Wnt ligands and signaling.

*Second, the primary function of Wg spreading is postulated to be stimulation of FSC divisions. The presentation of these measurements must be clarified and they must be repeated for conditional Wnt conversion. Accumulation of cells in stalks, encapsulation defects, arrested budding and so forth can potentially have many origins and are not sufficient to point the finger specifically at altered rates of FSC division. In Fig. 3 the authors report the total number of PH3+ cells in different locations. Crucial missing information is the number of cells evaluated in each region (to know the frequency of PH3 labeling). The most important category is labeled “Fas3-“ and discussed as FSCs. How exactly were these cells defined? How many were counted on average per germarium for controls and mutants (and, of course, total n values should be given for all categories for the purpose of evaluating statistics)? If the number of FSCs changed in different conditions the interpretation of results would be very different. It would also be good to have an additional measure of cell division (not least because PH3 staining is infrequent per cell and therefore difficult for small cell populations).
I am raising this point also because there are reasons to be suspicious of the conclusion that spreading Wg promotes division of FSCs and region 2b Fas3+ cells:-
(i) There is very little detectable Wnt signaling, measured by Fz3-RFP in 2b Fas3+ cells and hence it would be a surprise if that had a major direct effect on division rates (though I will admit that the phenotype could result from delayed effects of Wnt signaling in more anterior progenitor cells)
(ii) There are studies (Reilein et al., 2017) showing that supposed complete elimination of Wnt signaling (using a null arrow allele) does not greatly alter FSC division rates.
(iii) the authors prior work on this subject, which they cite, only directly measures PH3 labeling in “region 2” (and not on a per cell basis) for an Mmp2 mutation, which increases Wg spread but which may affect processes other than Wg spread (i.e. I do not find that the precedent of Wnt signaling affecting proliferation rates in and around FSCs is clearly established).*

In response to the reviewer’s request, we have expanded the FSC proliferation data to include the adult-conversion of *wg-> NRT-wg* (Fig. 3E), and no FSC divisions are observed in these germaria. We have clarified how we identify the region of the germarium containing follicle stem cells: we counted mitotic cells at the 2a/2b boundary, which generally had low but detectable fas3 staining and were anterior to the region of high fas3 staining. This is how we recognized them previously (Wang and Page-McCaw, 2014, as described in the methods), although the reviewer is correct that our Fig. 2C was poorly labeled. Further, these landmarks are used by many other labs to identify their location (Song and Xie, 2002; Nystul and Spradling, 2010; Hartman,O’Reilly, 2013; Laws and Drumond Barbosa, 2015.) Given the current conflict about where and how many FSCs exist in the germarium, our method would count follicle stem cells as described by both models. Both the Nystul camp (Fadiga 2020) and the Kalderon camp (Reilein 2017) agree that the FSCs are located at the 2a/2b boundary and anterior to the bright region of fas3 staining, with the main difference being how many cell diameters anterior to the boundary they extend. Our method would count only a subset of FSCs according to the Kalderon model – layer 1 but not layers 2 and 3 — but because layer 1 is more proliferative and gives rise to follicle cells, it is the most important layer to count. Further, counting this single layer avoids any possible contamination from mitotic germline cells, the other mitotic cell type in the germarium anterior to the 2a/2b boundary (or mitotic escort cells, if they exist, see Morris and Spradling 2011). Although FSC proliferation rates are low, we counted enough germaria to identify a total of 33 such positive cells in this region in controls, but we did not see a single pH3+ cell in this region in the various *NRT-wg* germaria. Unfortunately, we are unable to provide a denominator to the number of cells in each region, both because it is difficult to count these cells accurately, and further because there is no community agreement on how many FSCs are in this region. However, the cell density and general architecture of the *NRT-wg* and control germaria are similar, suggesting that we counted similar numbers of cells.

Realizing that we could not satisfy the reviewer with the data requested, we sought another way to address this question. We considered using FUCCI but decided that interpreting FUCCI would be no easier than interpreting pH3. The most rigorous experiment was to use a FLP-based mitotic recombination method to label FSC clones, as we did in our 2014 JCB paper; clone frequency is an indirect measure of proliferation, as recombination only occurs in cycling cells. The next paragraphs describe our efforts, which ended up not being useful. This work took about a year and is the main reason for the delay in resubmitting our revised manuscript.

Because the *NRT-wg* and control lines used in our work already contain *FRT* sites (see Fig. 1A), we originally though that FLP-based mitotic recombination methods would not be possible. However, in desperation we contacted JP Vincent and found that that they had *NRT-wg-HA* and control *wg-HA* lines that did not contain *FRT* sites. We acquired these lines and began building stocks to do 1) MARCM analysis and 2) the older technique from the Perrimon lab of reconstituting *LacZ* via *FRT*-mediated recombination of elements *X15-33* and *X15-29*. We were unable to build the necessary MARCM stocks. We were able to build stocks for the simpler *LacZ* system:

*hsFLP; wg^cx4^ X15-33/CyO*

*wg-HA X15-29 /CyO*

*NRT-wg-HA X15-29 /CyO*

When crossed and heat-shocked, we detected no (zero) clones from FSCs or GSCs in the following *NRT-wg* genotype:

$\frac{hsFLP}{+}$ ;$\frac{{wg}^{cx4} X15-33}{NRT-wg-HA X15-29}$

even though we observed germline and/or follicle stem cell clones in every germarium from controls:

$\frac{hsFLP}{+}$ ;$\frac{{wg}^{cx4} X15-33}{wg-HA X15-29}$

Despite the absence of clones, our PCR analysis clearly showed that the *X15-29* element was present on the *NRT-wg* chromosome. No clones were identified in more than 80 germaria over three experimental replicates. We used shorter recovery times to look for transient clones, expected to be plentiful, but saw none. We checked other organs, including the adult gut, and we even heat-shocked whole pupae to identify clones in any tissue; we never found clones in *NRT-wg/∆* but plenty in controls. In the course of doing these experiments, we discovered that the *NRT-wg/∆* flies were remarkably sensitive to heat-shock and died. Progeny of the following cross were heat-shocked for 1h at 37° as pupae close to eclosion, and then eclosed flies were counted:

*hsFLP; wg^cx4^ X15-33 /CyO	x	NRT-wg-HA X15-29 /CyO*

| **genotype** | **Number of adults observed** |
| --- | --- |
| *hsFLP/+; wg^cx4^ X15-33 /CyO* | 256 flies |
| *NRT-wg-HA X15-29 /CyO* | 282 flies |
| *hsFLP/+; wg^cx4^ X15-33/NRT-wg-HA X15-29* | 16 flies |
|  |  |

Results reported in Fig. 1B indicate that, without heat-shock, *NRT-wg/ wg^cx4^* flies are expected to represent ~18% of the total progeny. These results show that after heat shock and FLP induction, only 2.8% survive. Thus, it is possible the surviving animals we analyzed were selected to be resistant to FLP-mediated recombination. To investigate the survivors, we stained them for pH3; although they had no mitotic clones, they were positive for pH3 staining indicating that proliferation was not sufficient for FLP-induced recombination in the survivors. We do not include any of this data in the manuscript because we think it would be confusing, although we can include it in a supplement if so advised by reviewers or the editor.

Thus, our revised manuscript contains pH3 staining just anterior to the 2a/2b boundary as a measure of FSC proliferation. Although we wish we had a second method to validate this finding, our pH3 data is convincing nevertheless because of the large number of samples examined.

*Other details:
P8 Where were cysts dying? If cysts died at various locations (not just the oldest) one would expect several to mature and be abnormally large in the germarium. Is the idea that there is no budding or very little? It is still hard to reconcile a phenotype of normal morphology (including cyst maturation) with absolutely no FSC proliferation. It would be informative to look at wg-ts in the same way. Why might the phenotypes differ? What do the authors think is the origin of cyst apoptosis? It seems unlikely to be a direct response to Wg. What somatic cell defect might be the origin that differs in the two con*

We don’t share the reviewer’s expectation of abnormally large dead cysts in the germarium; our expectation is that dead cysts would be resorbed (probably by escort cells). 16-cell cysts were affected. The mechanism of cyst apoptosis is outside the scope of this study. It may be a response to the failure of FSC divisions, directly or indirectly, which makes it a downstream response to Wg. Unfortunately, we have not had success with the *wg^ts^* allele — it seems likely that it has picked up modifiers in the decades since it was identified.

*The conditional wg phenotype reported (p9) is not analyzed sufficiently (as stated earlier) but can the authors explain why the phenotypes that are reported are significantly different from those of the straightforward Nrt-wg allele?*

It is true that when NRT-wg is induced at different times (throughout life vs. in adults) we observe different mechanisms by which the germarium adapts to the lack of FSC proliferation. In adult-flipped flies, we observe enlarged germaria with excess germline cysts, whereas when NRT-wg is expressed from the start, we observe cell death. This difference must originate lies in development, and one easy possibility is that gut development is altered in NRT-wg flies, leading to mild starvation in addition to FSC non-proliferation. We discuss this possibility in the paper when we introduce the adult-onset conversation experiments.

*Summary
The NRT-wg allele provides a useful way (supplementing earlier studies with Dlp) to judge the role of Wg spreading in setting up the normal pattern of Wnt signaling and to investigate its role but I believe the main questions I have posed must be answered to fulfill that objective. The data presented suggest that the overall outcome will be positive but there are important details missing.*

**Reviewer #2:**

*This manuscript describes clear evidence that the export and movement of Wg over several cell diameters is required for proper function of follicle stem cells in the Drosophila ovary. This work will be of interest to researchers working in a variety of fields: fly oogenesis, stem cell biology, and Wnt signaling. In general, the data are nicely presented, with quantitation and beautiful imaging to support the authors' conclusions. One important conclusion from this work refutes an earlier paper from another lab, which claimed that Wg does not need to move from cell to cell to generate proper pattern in the Drosophila wing imaginal disc. The work presented here uses the same membrane-tethered Wg construct, and demonstrates nicely that in the context of the ovary, export and movement of Wg is essential. To me, this suggests that different tissues in the fly may have different capacities to handle this aberrant form of Wg. Wg is naturally membrane-tethered due to covalent attachment of a lipid moiety so it would not be surprising if cells in some tissues, such as the wing disc, can handle a form that is artificially tethered through an ectopic transmembrane domain. It might be worth adding a sentence or two to point this out, to try to reconcile these new data with the previous publication.*

Thank you for the suggestion. We have added this point to the next-to-last paragraph of the revised manuscript.

*The authors could also strengthen their case by being more explicit about the control construct that they are using. The methods section describes this control as the unflipped wg{KO; FRT Wg FRT QF}/CyO, which has a wg cDNA replacing the endogenous wg sequence. However, they give us no information about whether this chromosome homozygoses and if the homozygous vs. hemizygous state was being used as the control. Minimally, they need to explicitly state in the figure legend the genotype of their control flies, and they should include the eclosion data for this chromosome in their Fig. 1A, to ascertain whether this chromosome suffers the same problem as the NRT-wg chromosome in the homozygous state.*

This control chromosome is now diagrammed along with all others in new Fig. 1A, and we have clarified that it is homozygous viable in Fig. 1B. We have tried to clarify the genetics throughout the text – we have been very rigorous, and we hope it is more apparent now.

*They could also do a service to the Wg/Wnt field and address more rigorously the question of the NRT-wg homozygous phenotype. If the unflipped wg{KO; FRT Wg FRT QF}/CyO and wg{KO; FRT wg FRT NRT-wg}/CyO both show the expected eclosion rates for homozygotes, this would tell us that at least the parental chromosome used in the construction of the fly lines did not have an unrelated background mutation that would account for the reduced viability of the NRT-wg homozygotes. Indeed, if the wg{KO; FRT wg FRT NRT-wg} chromosome homozygoses and has normal germaria, then it would be possible for the authors to eliminate the possibility of a second, unrelated mutation causing the NRT-wg homozygous phenotype. There is a beta-tubulin-driven flippase available at the Bloomington Stock Center that drives in the male germline and flips FRTs at high efficiency. This could be used to swap NRT-wg into their control chromosome, providing a much cleaner reagent for their experiments. If instead these "control" chromosomes have the same problems as NRT-wg, as suggested by the presence of a balancer chromosome in the stock, then it is important to make clear that none of these lines can be used as homozygotes.*

We have taken the authors suggestions. Fig. 1B now shows that the unflipped *wg{KO; FRT Wg FRT QF}/CyO* and *wg{KO; FRT wg FRT NRT-wg}/CyO* both show the expected eclosion rates for homozygotes (columns 1 and 4). We got the beta-tubulin-driven flippase from Bloomington as suggested and converted *wg{KO; FRT wg FRT NRT-wg}* to *wg{KO; FRT NRT-wg}.* Columns 4 and 5 of Fig. 1B compare the homozygous viability of this chromosome before and after flipping. In panel C we took it one step further, and asked if animals carrying the flipped chromosome in trans to the unflipped chromosome show increased lethality, but their survival is very close to the expected Mendelian ratio. We appreciate the suggestions to increase the rigor of our study.

The authors also need to be cautious about their interpretation of the apparently restricted distribution of NRT-Wg in Fig. 2H. It is known that any mutation that disrupts export of Wg will produce such high accumulation of Wg in wg-expressing cells that in heterozygotes, the half-dose of normal Wg spreading from those cells cannot be detected [See for example van den Heuvel et al. (1993) EMBO J 12: 5293-5302]. If the authors were to stain germaria from wgts/CyO females, it is entirely possible that their Wg accumulation would look exactly like panel 2H but the ovaries would be completely normal. If the authors want to strengthen their case, they could include this experiment, and show us that they can detect the spread of Wg produced from the wild-type allele in wgts heterozygotes. I would also be interested to know if the NRT-wg/CX4 germaria show such a dramatic accumulation of Wg staining in the cap cells. Having 2 doses of the artificially tethered form might clog up the secretory apparatus, causing this intense staining, whereas having just one dose might allow the cells to handle a low level of cell to cell spreading. Indeed this might explain why the NRT-wg/CX4 germaria look so much better than NRT-wg homozygous germaria. Perhaps some of the tethered Wg is able to spread and induce proper cell fates at earlier stages of ovary development. This putative low-level spreading might just be insufficient to reach the follicle cells, resulting in the phenotypic effects shown by both homozygous and hemizygous NRT-wg.

Unfortunately, in our lab the wingless-ts allele does not behave as reported, possibly because it has accumulated modifiers, and for that reason we have not performed the experiment suggested here with wingless-ts. In general, we agree with the reviewer’s interpretation: our data support the conclusion that the tethered wingless is accumulating at such high levels that its function is impaired, as cutting it by 50% improves function. The reviewer is suggesting that the difference lies in clogging up the secretory apparatus, and we suggest the difference lies in increased juxtacrine signaling. Unfortunately, cutting the level by 50%, which we have done in several experiments, cannot distinguish between these models.

Minor comments:
1. The sentence "This study provides the clearest example of long-distance Wg/Wnt spreading." in the Author Summary seems like an overstatement. Perhaps it would stand the test of time better if it was qualified with a "to date" or "yet".

We have removed this statement from the summary.

2. In Fig 1, the authors state in the text that "9.7% of the male progeny were homozygous for NRT-wg and only 6.3% of the female progeny were homozygous for NRT-wg (Fig. 1A)" but the legend in the figure indicates that the genders are reversed. Which one is correct?

We have removed the information on sex of the escaper NRT-wg flies, as we did not find any significant sex-based difference, and it was an unnecessary distraction.

Have all data underlying the figures and results presented in the manuscript been provided?
Large-scale datasets should be made available via a public repository as described in the PLOS Genetics [data availability policy](https://na01.safelinks.protection.outlook.com/?url=http%3A%2F%2Fjournals.plos.org%2Fplosgenetics%2Fs%2Fdata-availability&data=02%7C01%7Candrea.page-mccaw%40vanderbilt.edu%7Cd03bcd18e96045e6463908d60c571938%7Cba5a7f39e3be4ab3b45067fa80faecad%7C0%7C0%7C636709965569378420&sdata=YyQnBVXv%2Bw%2BV8ZVMIYTSnCQEnORSPh9YEIX2vWV7XA4%3D&reserved=0), and numerical data that underlies graphs or summary statistics should be provided in spreadsheet form as supporting information.

Reviewer #1: No: I did not see any spreadsheets for the various graphs (I would not scrutinize them but if that is the policy....)

These spreadsheets are now provided.

Reviewer #2: Yes

--------

**Editor:**

Thank you very much for submitting your Research Article entitled 'Extracellular spreading of Wingless is required for Drosophila oogenesis' to PLOS Genetics. Your manuscript was fully evaluated at the editorial level and by independent peer reviewers. The reviewers appreciated the attention to an important problem, but raised some substantial concerns about the current manuscript. Based on the reviews, we will not be able to accept this version of the manuscript, but we would be willing to review again a much-revised version. We cannot, of course, promise publication at that time.

In looking at your manuscript and the reviewers' comments, I strongly agree with Reviewer 1's comments concerning the genetic backgrounds used.  Linked mutations are a big issue in Drosophila genetics, and it looks like you have one on the NRT-Wg chromosome.  NRT-Wg/CX4 transheterozygotes are the best way to control for this, and I encourage you to include that genetic background for all readouts examined.

Previously we had used NRT-wg/CX4 for viabililty, fertility, lifespan, and ovary size. In response to these commetns, now we have expanded the experiments on FSC and somatic cell mitosis (pH3 staining Fig. 3) and the analysis of apoptotic cysts in NRT-wg germaria (Fig. 4) to include NRT-wg/CX4.

Reviewer 1's feeling that NRT-Wg/+ is the appropriate "wild-type" control is more complicated in my view, at least when performing Wg immunostains (see Reviewer 2's insightful comment about Wg staining in wg[ts]/+ tissues).  The other additional experiments requested by Reviewer 1, e.g., the use of Fz3 and demonstrating that the proliferative state of FSCs in the adult replacement of wg for NRT-wg I also view as important.

These experiments were performed and results are shown in Figs. S1 and S2.

I also agree with Reviewer 2 that commenting and discussing the differences in between the ovaries and other tissues where NRT-Wg is sufficient for Wg function will help the readership better understand what this interesting allele teaches us about Wg signaling in flies.

As we noted to reviewer 2, this interpretation is now discussed in the second-to-last paragraph.
