# Supplementary material for: Extracellular spreading of Wingless is required for Drosophila oogenesis
Source: PLoS Genet. 2021 Apr 2;17(4):e1009469. doi: 10.1371/journal.pgen.1009469 (PMC8046344; doi:10.1371/journal.pgen.1009469)
Supplement: Attachment — Submitted filename: Response to reviewers 2-15 21 IW.docx [file pgen.1009469.s005.docx]

Response to Reviewers

*Reviewer #1: This manuscript uses a membrane-tethered form of Wingless to address how spreading of an extracellular morphogen influences follicle stem cell proliferation in the fly ovary. This is a nice follow-up of earlier work from the same lab showing that Dally-like Protein is essential for follicle stem cells, presumably through its role in regulating extracellular Wingless. The results presented here not only establish the importance of Wingless protein spreading to the follicle stem cells, but also reveal problems with the original membrane-tethered Wingless construct. In addition, the authors have found interesting complexities in the use of Fz3-RFP as a reporter of Wingless activity. These observations will be of value to the Wnt field and the fly community. The revised manuscript reflects a great deal of effort to address the concerns of the first set of reviewers, and I believe that the concerns have been addressed as well as is biologically possible.*

We thank the reviewers for their positive evaluation.

*Reviewer #2: I would like to start by commending the other reviewers for their initial comments, and the authors for responding earnestly and with a manuscript that is generally very careful in presenting data, logic and conclusions.
Although I did not see the first version of the manuscript, I find that reviewer 1 has mentioned numerous key issues regarding the ovariole and FSC biology that I fully endorse as crucial. Both reviewers brought up critical genetic issues, and reviewer 2 broke the ice with regard to thinking beyond Nrt-Wg simply behaving as unable to signal at a distance. I don’t think any of these issues are fully resolved despite great efforts from the authors, and that a couple of key issues mean that the conclusions offered, at least as phrased, are not really supported.*

*The biggest elephant in the room is that animals with only Nrt-Wg as a genetic source of Wg in the ovary have a Fz3-FP pattern that suggests more Wnt signaling in and around FSCs rather than less. That situation would be entirely consistent with the observed reduction in cell division because others have observed that strongly increased Wnt signaling greatly reduces EdU incorporation in marked FSC clones (Reilein et al., 2017; Melamed & Kalderon, 2020).*

We agree with the reviewer that increased Wnt signaling around the FSCs would lead to reduced divisions, and in the previous version, we intended to imply that model in the last paragraph of the Discussion. Now we make it explicit, that the increased Wnt signaling inferred from Fz3-RFP is consistent with the loss of FSC proliferation.

Unlike the reviewer, we do not put as much stock in the Fz3-RFP results, as we discuss below.

It also becomes plausible that early FCs, which normally experience almost no measurable Wnt signaling, could have a proliferation deficit (because Wnt pathway activity is increased, not decreased).

Although this model is plausible, our data show that there are fewer divisions of cells in region 2b (Fig. 3), which does not support this model.

*The authors likely do not favor this interpretation because it appears to conflict with their conclusions from a 2014 study. That study mainly relied on indirect measures of cell division rates, did not make strict measurements per FSC, and examined situations (altering Dlp and Mmps) where the Fz3-RFP reporter was not quantified specifically in FSCs but appears to be altered quantitatively far less than the increases reported in the current work. The inhibition of EdU incorporation that was observed by others in axn or apc mutant cells corresponds to a large increase in Wnt pathway activity in FSCs. It seems quite possible that the smaller increase seen for Mmp mutants has different proliferative consequences to the large increases due to axn, apc and Nrt-Wg in this study. It has already been shown that Wnt signaling also has a positive influence on EdU incorporation under specific conditions (when JAK-STAT signaling is reduced) (Melamed & Kalderon, 2020).*

We thank the reviewer for the effort to synthesize our current findings with our published findings.

*Since the Fz3-RFP result is an important phenotypic intermediate, and the authors described significant difficulty in generating rare appropriate recombinants, it would be useful (though perhaps not necessary) to examine proliferation in the very same animals (or genotypes) used for Fz3-RFP measurement.*

As the reviewer suggests, it is not necessary for us to analyze proliferation in *fz3-RFP, NRT-wg* flies -- we already present pH3 data for three different *NRT-wg* containing genotypes, with similar results in each.

*How does the observed increased Wnt signaling over the anterior 2/3 of the germarium come about?*

This is certainly an interesting question, and we have two models. One is that there is compensation by other Wnt ligands when insufficient Wg spreads posteriorly. The other is that excess Wg near the cap cells indirectly turns on the Fz3 reporter, perhaps as part of a relay turning on other Wnt ligands. The key to distinguishing them would seem to be the fact that *NRT-wg/CyO* (with one wild-type *wg* allele) has increased *fz3-RFP* compared to wild-type. However, we note that heterozygous wild-type Wg does not seem to spread nearly as far as homozygous wild-type (Fig. S1 in Wang et al), so these data cannot fully distinguish between these models. A third possibility is that the Fz3-RFP reporter responds to other non-Wnt signaling. In Wang et al, we tested the specificity of Fz3-RFP for Wnt signaling by overexpressing *axin* or *arm^S10^*, and we found it to be turned off or turned up in these conditions, respectively. But in the *NRT-wg* condition there is some kind of altered signaling, and we cannot exclude the possibility that another signaling pathway turns on the reporter under these conditions. Teasing out these mechanisms is the subject for another study.

*The authors say that direct Wg staining shows it is more restricted. The supporting data (Fig. 2G, H) do not appear to me to be very sensitive tests (would also be better for the allele over a null because that was the most informative phenotype for other properties) and it is always possible that details of fixation and staining may accentuate some Wg populations over others (not to mention that the actual molecules involved in signaling may be too few to detect). It is possible that Wg, and even Nrt-Wg, can travel in a variety of ways (exovesicles, cytonemes, transcytosis, lipoprotein particles etc.) and be released from various lipid associations at a variety of points by proteases. I personally would not feel assured from the data presented that Nrt-Wg itself is not spreading.*

The question of whether NRT-Wg can spread is central to our paper. In their 2014 Nature publication, Alexandre and colleagues provide evidence that NRT-Wg is indeed membrane tethered. Total Wg staining showed that NRT-Wg was restricted near the dorso-vental boundary in wing imaginal discs (and similar evidence is provided by us in the *NRT-wg* germaria with the exception that our staining protocol detects only extracellular Wg). Additionally, Alexandre et al show that no NRT-Wg staining is detected in small patches of cells where NRT-Wg is excised using Flp-mediated recombination (their Fig. 1I). These data suggest that NRT-Wg expressed in the cells abutting the ‘NRT-Wg excised’ cells fails to spread to the excised cells arguing against cleavage/exosome/lipoprotein particles/transcytosis-mediated spreading of NRT-Wg. Moreover, the Wg-responsive gene Distal-less is not expressed in *wg^CX4^* clones generated in NRT-Wg background (Fig. 2B). In contrast, Distal-less expression is maintained in *wg^CX4^* clones generated in wild-type background (Fig. 2A). These data show that wild-type Wg can spread from neighboring cells to induce Distal-less expression in *wg^CX4^* clones but NRT-Wg fails to function similarly.

For our anti-Wg staining, we did not examine NRT-wg/null germaria as requested by the reviewer because they would be difficult to interpret. As we showed in Fig. S1 of Wang et al 2014, Wg protein levels are significantly lower in heterozygous wg (wild-type/null) flies, and it would be difficult to distinguish changes in localization in *NRT-wg/null* because of the reduced levels.

As the reviewer indicates, it is always possible that the threshold of ligand necessary for signaling is below the visible level – and that consideration means that no staining result can ever fully satisfy this question. However, in our study, we look to function: NRT-Wg does not behave like Wg, in all the assays that we show throughout the paper. We checked very carefully that these phenotypes are caused by NRT-Wg and not another locus on the chromosome. We observe functional changes caused by NRT-Wg, and we observe a concomitant visible change in localization consistent with the model that it is indeed tethered. If the reviewer does not believe that the tethering is causing the functional change, then what is? We feel confident that the phenotypes we characterize throughout the paper, including the FSC proliferation defect, are caused by a lack of Wg spreading.

*The authors suggest that Fz3-RFP may be increased indirectly. That is also plausible but potentially extremely complex (but is not obviously a result of loss of normal Wg spreading because it is seen for Nrt-Wg/CyO too).
It may well not be practical but the only resolution I can imagine to the question of whether Nrt-Wg itself has a shorter or longer range is to eliminate the other Wnts simultaneously.*

Interestingly, the graph in Fig. S2 shows that the more Wg there is near the cap cells (with wild-type *wg* providing some, and *NRT-wg* providing a lot), the more Fz3-RFP signal is observed. Two copies of *NRT-wg* seem to have an increase over just one. These data may suggest that Fz3-RFP signal is triggered by a relay of Wg signal near the cap cells, one of the models discussed above. We agree with the reviewer that it is not practical to eliminate all other Wnts simultaneously.

*At present, I think the statement that Nrt-Wg is tethered and acts only at short-range is only an assumption, with the Fz3-RFP results seriously challenging the assumption. My opinion is therefore that the central conclusion that includes that assumption cannot be supported. I do, however, believe that if the study were titled as an exploration of Wg range, and conclusions, were appropriately tempered on this key point, that there is a lot of useful exploration here (and that apparent contradictions between Wnt signaling and proliferation are resolved by taking the Fz3-RFP quantitative data at face value).*

We respectfully disagree with the reviewer about the importance of the Fz3-RFP reporter data. As we showed in Fig S.1, it is not specific for Wg, and under mutant conditions, we cannot be certain it remains specific for Wnt signaling. For the reasons described above, we are confident that NRT-Wg is not spreading, and although we cannot connect all the dots for why the FSCs are not dividing, we are confident it derives from a lack of Wg spreading.

*In my view, the Nrt-Wg studies do not resolve the issue of which Wnts are “more important” for directly signaling to FSCs in the germarium (and they add useful information for those principally interested in the subject of long-range Wg signaling in various tissues without reaching a definitive conclusion).*

We agree – our study indicates that Wnt signaling is highly complex.

*I think that if the authors highlighted the quantitative results they found for Fz3-RFP (especially if they focus on the FSCs immediately anterior to the Fas3 border) and reducing various sources of Wnts, they could make the case that both Wg from cap cells and Wnts (mainly Wnt4) from Escort cells (collectively) contribute to the normal Wnt signaling pattern. Since it is already clear that quantitative levels of Wnt signaling affect a variety of FSC behaviors, it is to be expected that the quantitative changes observed are likely functionally significant. I believe that presentation would largely resolve prior publications on the subject and be cited as such in the future.*

We incorporated the reviewer’s suggestion by ending the Fz3-RFP section as follows: “We conclude that Fz3-RFP is a complex reporter for many types of Wnt signaling, and that the Wnt signaling pattern in the germarium indicated by this reporter is the sum of signaling from several ligands.

*Some other issues:*

*1. Please write out actual genotypes relevant to all data panels. Despite Fig. 1 and clarification of some specific questions previously raised by reviewers, there were two or three places where I was unsure of the genotype.*

We have included a new section of the Materials and Methods with a complete genotype for every figure panel and graph.

*2. I was unsure whether flp-out events in certain cases were just assumed or actually tested.*

We assessed the germline conversion to *wg* from *NRT-wg* by PCR. Experiments with adult-onset somatic conversion to *NRT-wg* were assumed (3E, 4H), as these events are difficult to assess. There is no reason for doubt, however, as all data were consistent with the germline expression of *NRT-wg*.

*3. Like reviewer 1, I remain surprised that there can be normal ovariole morphology with no FSC division. Does the sequence of egg chamber maturation indicate arrest or extremely slow progress?*

We are also surprised, but after receiving reviewer 1’s feedback, we analyzed a third *NRT-wg* genotype and found the same result (Fig. 3E). Looking at the ovarioles, we cannot distinguish between arrest or extremely slow progress, but considering the fertility data, it would seem that the maturation rate is just extremely slow.

*4. The data for Fz3-RFP in Fig. S1 seem to suggest Wnt4 is a particularly strong contributor. If correct, the text should reflect that and other appropriate quantitative impressions (most readers will not look at these data).*

We now report the levels of reduction RFP fluorescence in the main text, in response to the reviewer’s suggestion. We also note that RNAi lines may have different levels of efficacy, so we do not want to make too big a deal of these data.

*It is stated that for Nrt-Wg that Fz3-RFP is higher in escort cells. That is mis-leading. It is clearly higher also in FSCs and beyond. Was Fig. S2 made with Fas3 staining- ideally that should be included but I am fairly sure from the images that Fz3-RFP extends beyond escort cells.*

Without Fas3 staining, we cannot say for sure, nor is it obviously true for *NRT-wg/null*. To alleviate the reviewer’s concern, we now state that “RFP levels were unexpectedly increased throughout the anterior germarium”.

*5. The reference to previous measures of FSC mitotic clone induction rate and attempts to repeat that are, in my opinion, problematic. Those induction rates are very variable amongst animals and batches using uniform conditions and what is observed after several days depends more on the competitive nature of the FSC than the frequency of initial marking. (It is also not necessarily true that the X15-29 system requires mitosis [and not obvious that it would], according to Kirilly & Xie). I would not advise including the unpublished results distributed to reviewers.*

We will not include these data in the final manuscript, as suggested by the reviewer.

6. *I agree with reviewer 1, that PH3 staining frequency is really only informative if on a per cell basis. However, I understand the associated difficulties and for the major point being made, I think it is clear that Nrt-Wg animals have reduced PH3 labeling, though the quantitative nature of the deficit remains unknown. I remain surprised that the authors do not use EdU incorporation as a simple, albeit expensive, secondary measure (no one of these methods actually necessarily report division rates but they can both be compared to other previously measured genetic conditions).
The identity of the cells measured is important.
I would agree with the authors that useful and reliable data can be gained from measuring all cells immediately anterior to strong Fas3 staining. Even if the authors, reviewers or readers were still to believe the out-dated dogma of two FSCs, there is no way for those cells to be distinguished from the other 6 or so cells residing in exactly equivalent locations (no known marker differences; of course, entirely compatible with them all being equivalent), so counting all layer 1 FSCs (Reilein et al., 2017) will capture all “original-dogma” FSCs. My own preference would be for all FSCs to be included but I know that is technically harder to do. I also believe that doubt about conclusions from Reilein et al., 2017 should only be promulgated if the authors have an objective, named basis for doubt after careful reading of the relevant papers, including the BioRxiv deposition from the Kalderon lab.*

We did not intend to raise doubts about conclusions from the Reilein paper. We do acknowledge, however, that this is an area where there is disagreement, and the existence of disagreement is a fact. Our interest is in convincing the reader that our FSC proliferation results are valid. We have amended our language somewhat, to refer to the Reilein 3-ring model as the “current model” and the Nystul 2 FSC model as the “older model”.

*The authors are incorrect in quoting Fadiga & Nystul with regard to FSC location, and in saying in response to reviewers that all studies place FSCs anterior to the strong Fas3 border. Fadiga & Nystul place them one cell further posterior (making the weak vs strong Fas3 border very clear, and the same as designated by others). Moreover, in Rust et al., the authors assert that all FSCs express Fas3 based on the Fadiga “result”. The alleged positioning of FSCs in those studies is indeed contrary to all prior studies, including several from the senior author and previous studies from the authors of the current study. The simplest solution is to eliminate the Fadiga citation because it does not support the (correct) assertion.*

We are now careful about not using the Fadiga citation to support this claim in the new version.

Editor: In reading through the detailed reviewer's comments, there are several additional experiments that are suggested, along with many questions/concerns about the interpretation of your results.  I don't think additional data are required for you to address the reviewer's points.  But I ask that you carefully consider the reviewer's concerns, and address them thoroughly in the rebuttal.  In addition, I ask you to modify the text of your manuscript where appropriate to take the reviewer's views into account.

One additional comment about the fz3-RFP reporter: I feel that both you and the reviewer give this reporter too much credit as an accurate measure of Wnt signaling.  RFP expression is driven by an endogenous stretch of regulatory DNA that may respond to inputs in addition to Wnt/beta-catenin signaling.  The reviewer feels the increase in fz3-RFP in NRT-wg backgrounds (including NRT-wg/CyO - do those flies have normal patterning and fertility?) suggests that NRT-wg/null is similar to Axin or Apc clones.  That seems unlikely and we don't have a direct comparison, i.e., fz3-RFP expression in those mutant backgrounds.  I agree with your view that the fz3-RFP reporter is complex, and this complexity may involve more than Wnt signaling (at least in a NRT-wg background).

We thank the editor for clear guidance on how to move forward with this manuscript. With respect to the Fz-RFP reporter, we note that our faith in its specificity for Wnt signaling comes from previous control experiments reported in Wang et al, 2014 (Page-McCaw lab), and in Dae et al, 2017 (Montell lab). Although it is possible it may lose specificity in some mutant backgrounds, we feel that introducing that caveat is too speculative and too hard to discuss in the body of this paper.
